# Numerical Investigation of the Effect of Stenosis Geometry on the Coronary Diagnostic Parameters

**DOI:** 10.1155/2014/354946

**Published:** 2014-09-01

**Authors:** Sarfaraz Kamangar, Govindaraju Kalimuthu, Irfan Anjum Badruddin, A. Badarudin, N. J. Salman Ahmed, T. M. Yunus Khan

**Affiliations:** ^1^Department of Mechanical Engineering, University of Malaya, 50603 Kuala Lumpur, Malaysia; ^2^Faculty of Engineering & Technology, Multimedia University, 75450 Bukit Beruang, Malacca, Malaysia

## Abstract

The present study deals with the functional severity of a coronary artery stenosis assessed by the fractional flow reserve (FFR). The effects of different geometrical shapes of lesion on the diagnostic parameters are unknown. In this study, 3D computational simulation of blood flow in three different geometrical shapes of stenosis (triangular, elliptical, and trapezium) is considered in steady and transient conditions for 70% (moderate), 80% (intermediate), and 90% (severe) area stenosis (AS). For a given percentage AS, the variation of diagnostic parameters which are derived from pressure drop across the stenosis was found in three different geometrical shapes of stenosis and it was observed that FFR is higher in triangular shape and lower in trapezium shape. The pressure drop coefficient (CDP) was higher in trapezium shape and lower in triangular model whereas the LFC shows opposite trend. From the clinical perspective, the relationship between percentage AS and FFR is linear and inversely related in all the three models. A cut-off value of 0.75 for FFR was observed at 76.5% AS in trapezium model, 79.5% in elliptical model, and 82.7% AS for the triangular shaped model. The misinterpretation of the functional severity of the stenosis is in the region of 76.5%-82.7 % AS from different shapes of stenosis models.

## 1. Introduction

Coronary artery disease (CAD) is one of the leading causes of myocardial infraction in human, due to the development of atherosclerotic plaque on the inner side of the wall of arteries. It brings the most effective changes in pressure, velocity, wall shear stress, and impedance on the blood flow [1]. The flow patterns such as velocity directions strongly influenced by the geometry of the stenosis formed and it is more complex to assess the physiological severity of an intermediate stenosis in a single vessel or branched vessel using usual coronary angiogram or multislice computed tomography [2, 3]. The true functional severity of coronary artery stenosis is assessed by pressure drop and flow [4–6].

However, the functional significance of stenosis is generally measured by the diagnostic parameters FFR [7] (FFR; the ratio of maximum blood flow in a stenotic artery to maximum blood flow if the same artery was normal) and coronary flow reserve [8] (CFR; ratio of hyperemic flow to the flow at resting conditions). Many of the clinical studies show that a FFR value of ≤0.75 identifies ischemia-causing coronary stenosis with an accuracy of 90% [6].

FFR is clinically well proven diagnostic parameter [6, 7]. In the presence of stenosis, a cut-off value of FFR < 0.75 is almost able to induce myocardial ischemia, whereas FFR > 0.8 never associated with exercise-induced ischemia in a single vessel coronary artery disease (CAD) as evident from the numerous clinical trials [6, 8, 9] which indicates that the gray zone for FFR is between 0.75 and 0.80 [3] that falls under the intermediate area stenosis (AS) (AS = area of the blockage due to stenosis/area of the lumen, free from stenosis). The functional diagnostic parameter FFR is performed with 0.014 inch diameter intracoronary pressure wire to record the distal pressure under hyperemic condition, which is induced by microvascular vasodilator, adenosine, or papaverine [6].

Plenty of research concentrating on the grey zone reported that the FFR value has been recorded with variation due to the insertion of guide wire [10] and downstream collateral flows [11] and by some other factors such as microvascular resistance, aortic and coronary outflow pressure [12], porous arterial wall [13], and blood flow through arterial wall compliance, and plaque characteristics significantly affect the FFR value [14] thus giving rise to the misinterpretation of the functional severity of the stenosis in the grey zone. It is also reported that the length of lesion and diameter are important geometrical variables which significantly affect the FFR. The pressure loss accompanying the viscous friction is proportional to the flow and is therefore directly proportional to the length of narrowing and inversely related to the fourth power of lumen diameter. Thus, as compared with length of lesion, stenosis diameter has a greater impact on distal coronary pressure or flow [15].

Many experimental, analytical, and computational simulations analyses on hemodynamic in stenotic arteries and computing stenosis severity were reported by many researchers in an axisymmetric stenotic straight tube [14, 16–20]. But the physiological significance of shapes of stenosis on diagnostic parameters is lacking as evident from the open literature. The present study includes 3D computational models of different shapes of stenosis on the diagnostic parameters (FFR, CDP, and LFC) for the given percentage area stenosis.

## 2. Method

### 2.1. Stenosis Geometry

**Figure 1:**
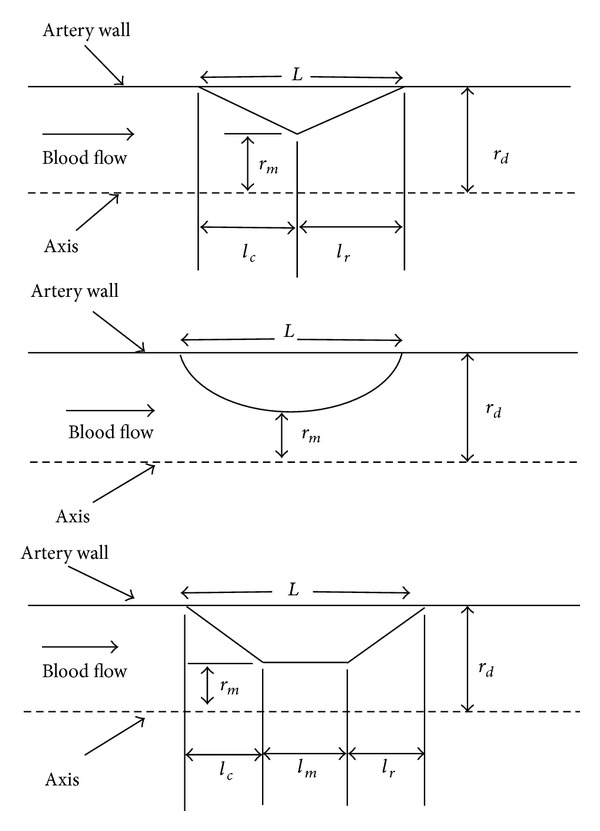
Schematic diagram for triangular, elliptical, and trapezium lesion geometry.

According to clinical data, stenosis does not have particular shapes [17–19]. Hence in the present study an attempt has been made to address the effect of possible shapes of stenosis on the diagnostic parameters for 70% (moderate), 80% (intermediate), and 90% (severe) AS.  Figure 1 depicts the different geometries of the stenosis considered. The triangular shape stenosis consists of converging (of length *l*_*c*_) and diverging (of length *l*_*r*_) sections, whereas trapezium model has converging (of length *l*_*c*_), throat (of radius *r*_*m*_ and length *l*_*m*_), and diverging (of length *l*_*r*_) sections. Moreover, proximal and distal radius is assumed to be identical (of length *r*_*d*_), and the length of the stenosis in all the model was fixed to 10 mm.  Table 1 shows the dimensions used for the triangle and trapezium to develop the models of stenosis considered in this study.

The elliptical shape stenosis model was developed by using the following equation [20]:

(1) $$\begin{array}{c} \frac{\tilde{\eta }\left(\tilde{z}\right)}{a}=1-\frac{h}{a}\sin\pi \left(\frac{\tilde{z}-d}{L}\right),\;d\leq \tilde{z}\leq d+L, \end{array}$$

where $\tilde{\eta }(\tilde{z})$ is the radius of stenosis, *a* is the radius of an artery, $\tilde{z}$ is along the axis of the artery, and *h* is the maximum projection of the stenosis into the lumen

(2) $$\begin{array}{c} \text{Area}\;\text{stenosis}\;\left(\text{AS}\right)\%=\frac{\left(\pi \times a^{2}\right)-\left[\pi \times {\left(a-h\right)}^{2}\right]}{\pi \times a^{2}}. \end{array}$$

### 2.2. Computational Modelling

Blood fluid is assumed to be non-Newtonian, incompressible, and governed by the Navier-Stokes equations:

(3) $$\begin{array}{c} \rho \left(\frac{\partial v}{\partial t}+v\cdot \nabla v\right)=\nabla \cdot \tau -\nabla P. \end{array}$$

The continuity equation for incompressible flow is

(4) $$\begin{array}{c} \nabla \cdot v=0. \end{array}$$

Here *v* = three-dimensional velocity vector, *t* = time, *ρ* = blood density, P = pressure, and *τ* = stress tensor.

The governing equation for the non-Newtonian and Bird-Carreau model is given by

(5) $$\begin{array}{c} \mu =\mu_{\infty }+\left(\mu_{0}-\mu_{\infty }\right){\left[1+{\left(\lambda \gamma \right)}^{2}\right]}^{(n-1)/2}, \end{array}$$

where *λ* (time constant) = 3.313 s, *n* (power law index) = 0.3568, *μ*_0_ (low shear viscosity) = 0.56 P, *μ*_*∞*_ (high shear viscosity) = 0.0345 P, and the density of the blood (*ρ*) is assumed to be 1050 kg/m^3^ [11]. A finite volume software CFX14.0 (ANSYS inc.) was used for flow simulations.

### 2.3. Boundary Conditions

**Figure 2:**
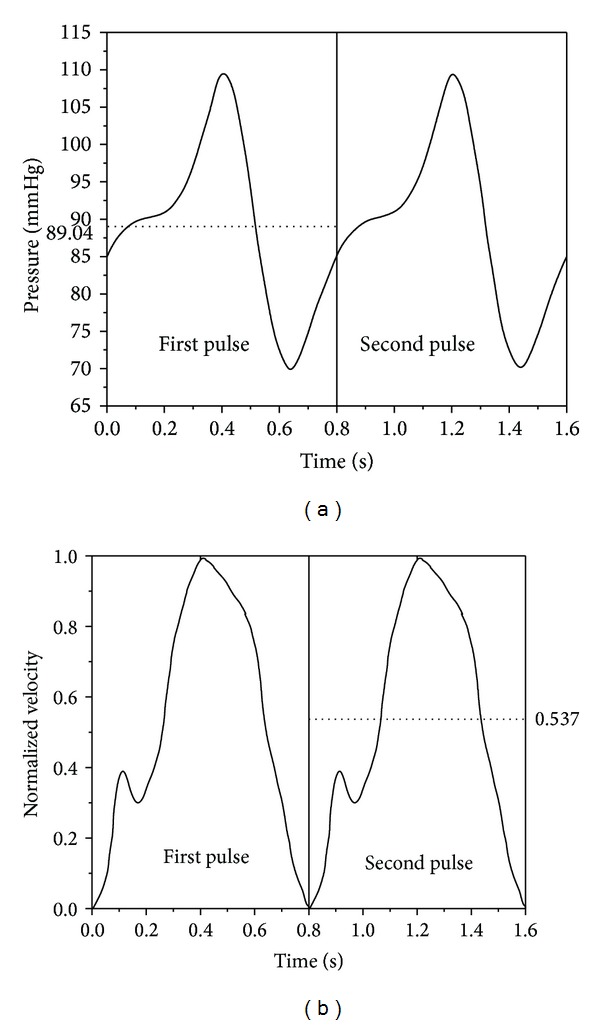
(a) Time varying physiological pressure applied at the inlet [14] and (b) coronary flow wave form $\overset{-}{u}/{\overset{-}{u}}_{p-t}$ versus *t* [10, 16]. The peak velocity ${\overset{-}{u}}_{p-t}$ corresponds to a normalized velocity of 1.0 so that the ratio of mean to peak velocity $\overset{-}{u}/{\overset{-}{u}}_{p-t}$ is 0.537.

In order to represent the realistic physiological conditions, the 3D numerical simulation was considered with a transient pulsatile pressure *p*(*t*) (Figure 2(a)) at the inlet and transient parabolic velocity *u*(*t*) at the outlet (Figure 2(b)) [14] with no slip condition at the arterial wall. All the three models were solved with the same inlet and outlet boundary conditions. The velocity profile for 70%, 80%, and 90% AS was obtained from the mean hyperemic flow rate $\left(\tilde{Q}\right)$ 175 mL/min, 165 mL/min, and 115 mL/min, respectively [14]. Under hyperemic flow condition, there could be possibility of instabilities in the flow because of the disturbances in cardiac pulse and irregularities in lesion anatomy in all the three models [14, 21, 22]. This condition could make the flow turbulent. Shear stress transport (SST) turbulence model which belongs to *k*-*ω* model family was employed in modelling which is more accurate and robust for low Reynolds turbulence computations [23, 24]. Initially, steady-state flow analysis was performed. This was followed by transient flow analysis considering the results from the steady-state analysis as the initial guess in CFD simulation.

For the steady-state analysis the following values of parameters at the inlet and outlet were taken, namely,

1. mean physiologic pressure at the inlet: 89.04 mmHg,
2. mean velocity at the outlet: 0.413 m/s, 0.389 m/s, and 0.271 m/s corresponding to 70%, 80%, and 90% AS.

### 2.4. Methodology

**Figure 3:**
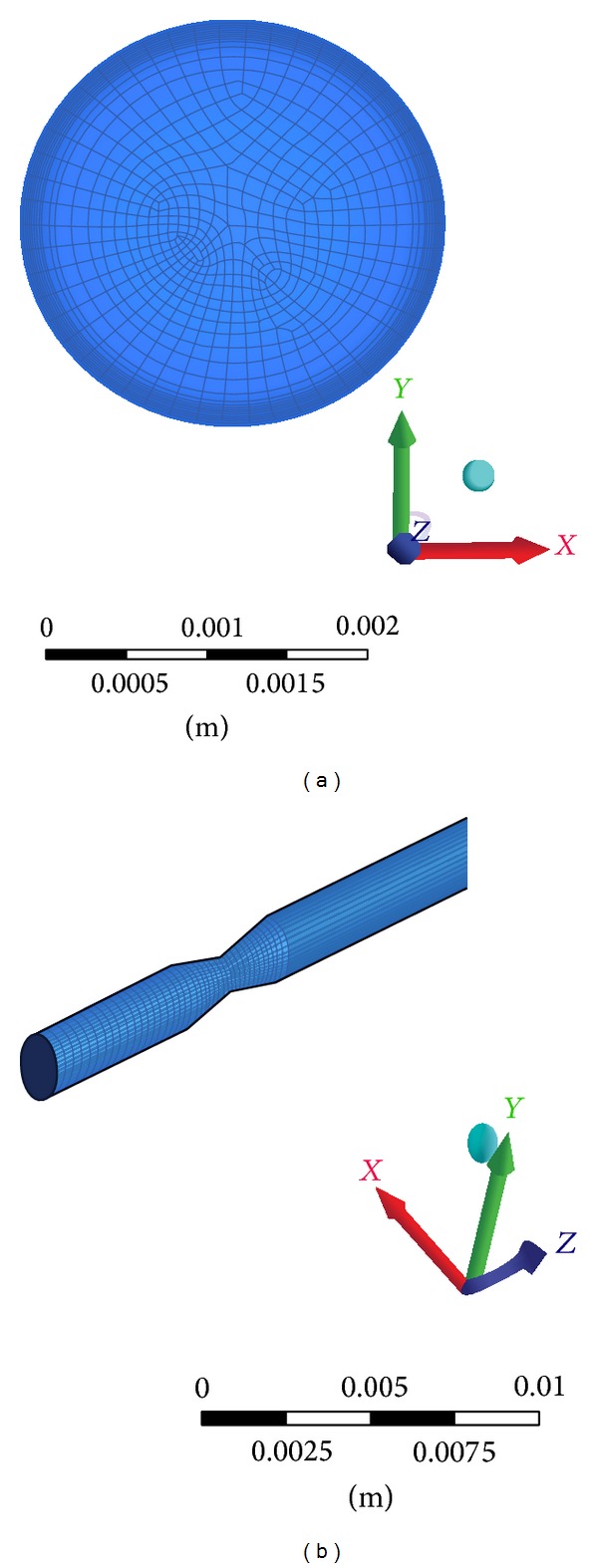
Computational mesh used for numerical study in the triangular model.

The 3D computational domains were initially discretized into elements in the range of 250,000 and 5,00,000 for 70%, 80%, and 90% AS for all the three different shapes of models with hexahedral meshes. The computational meshes are as shown in Figures 3(a) and 3(b). Commercially available software CFX 14.0 (ANSYS CFX, Canonsburg, PA) was employed for blood flow simulation. Furthermore, a mesh independent study was carried out with the elements in the range of 5,00,000 and 7,00,000 to ensure that computed velocity values differed by less than 0.3%. The transient flow analysis was run for 640 time steps (0.005 s per time step) representing 4 cycles (0.8 s each) of pulsatile flow with each time step converging to a residual target of 1 × 10^−5^. In all cases, without guide wire condition was considered.

## 3. Diagnostic Parameter

### 3.1. Fractional Flow Reserve (FFR)

At hyperemia, FFR is defined as the ratio of distal coronary pressure to aortic pressure [3, 6]:

(6) $$\begin{array}{c} \text{FFR}=\frac{{\tilde{p}}_{d}-{\tilde{p}}_{v}}{{\tilde{p}}_{a}-{\tilde{p}}_{v}}, \end{array}$$

where ${\tilde{p}}_{a}$ is the time averaged aortic pressure (mmHg), ${\tilde{p}}_{d}$ is the time averaged distal stenotic pressure (mmHg) measured at the end of pressure recovery [14], and ${\tilde{p}}_{v}$ is the venous pressure which is assumed to be 0 mmHg.

### 3.2. Pressure Drop Coefficient (CDP)

At hyperemia, CDP is a dimensionless functional parameter derived from fluid dynamics principles by considering time averaged pressure drop ($\Delta \tilde{p}$) and the velocity proximal to the stenosis [11, 12]:

(7) $$\begin{array}{c} \text{CDP}=\frac{\Delta \tilde{p}}{0.5\rho U_{a}^{2}}, \end{array}$$

where $\Delta \tilde{p}=({\tilde{p}}_{a}-{\tilde{p}}_{d})$ (N/m^2^) and *U*_*a*_  is the proximal velocity (m/s). CDP associates both viscous loss and loss due to momentum change in the flow across the stenosis.

### 3.3. Lesion Flow Coefficient

Banerjee et al. [16] developed normalized and dimensionless functional diagnostic parameter lesion flow coefficient (LFC) by considering the functional endpoints and the geometric parameters. The LFC ranges from 0 to 1 and it is the ratio of percentage AS and the square root of CDP evaluated at the site of the stenosis:

(8) $$\begin{array}{c} \text{LFC}=\frac{\text{percentage}\;\;\text{AS}}{\sqrt{\Delta \tilde{p}/0.5\rho U_{\left(a-h\right)}^{2}}}, \end{array}$$

where *U*_(*a*−*h*)_ is the velocity at the site of the stenosis (m/s).

## 4. Results

### 4.1. Time Average Pressure Drop in All the Models

**Figure 4:**
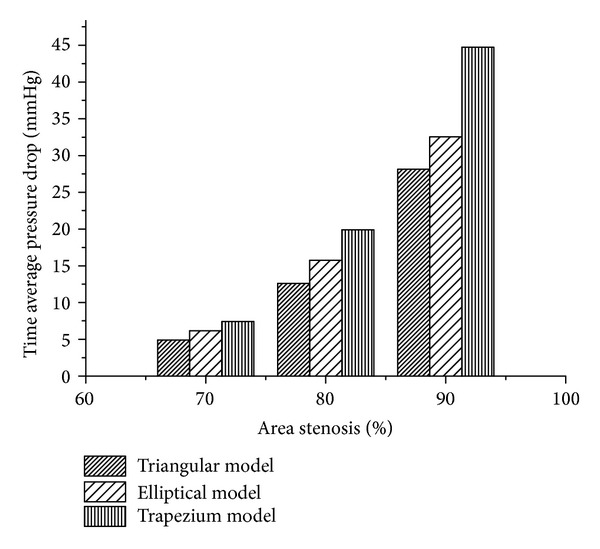
Bar graph showing variation of time averaged pressure drop across a given area stenosis with different shape stenosis (triangular, elliptical, and trapezium).

Figure 4 shows time average pressure drop in the triangular, elliptical, and trapezium models. The $\Delta \tilde{p}$ for trapezium shape stenosis was higher than the other two models and is followed by the elliptical and triangular shapes of models for a fixed stenosis severity. The $\Delta \tilde{p}$ increases in nonlinear manner as percentage AS increases for all the models. This could be characterized by the nonlinear nature of momentum changes on account of area constriction and vary with a second power of flow rate [14]. For triangular shape stenosis model, the pressure drop for 70% AS was 4.89 mmHg; however as the percentage AS increased from 70% to 80%, $\Delta \tilde{p}$ increased by 7.71 mmHg whereas from 80% to 90% AS, $\Delta \tilde{p}$ increased by 15.53 mmHg. In case of elliptical model, for 70% AS the drop in pressure was 6.17 mmHg from 70% to 80% increased by 9.58 mmHg whereas from 80% to 90% $\Delta \tilde{p}$ increased by 16.78 mmHg. Similarly for trapezium models pressure drop for 70% AS was 7.43 mmHg; $\Delta \tilde{p}$ increased by 12.45 mmHg as there was increase in stenosis severity from 70% to 80% AS and further increased by 24.85 mmHg as stenosis severity increased to 90%.

### 4.2. Effect of Shapes of Stenosis on Diagnostic Parameters

#### 4.2.1. FFR for All the Three Models (Triangular, Elliptic, and Trapezium)

**Figure 5:**
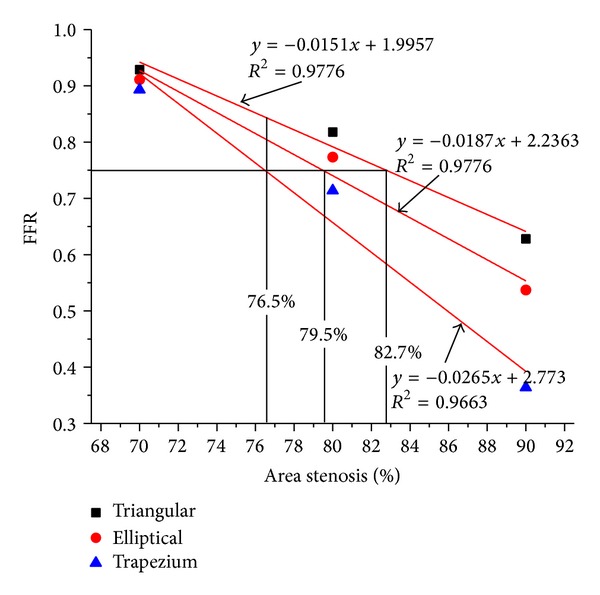
Variation of FFR values with different shapes of stenosis (triangular, elliptical, and trapezium).

The value of FFR for all the models decreases with the increase in the percentage AS. The FFR for the different shapes of models obtained in this study are in close agreement with available numerical results reported by Konala et al. [14] without guide wire. Konala et al. [14] have considered only trapezium model with a little change in geometry of stenosis. The computed values of FFR were plotted for the best fit approximation with linear correlation *R* ^2^ = 0.97. A horizontal line was drawn at FFR = 0.75 which represents the cut-off value to determine a range of AS with possible misdiagnosis as shown in Figure 5. This horizontal line intercepted the FFR—AS lines intersect at 76.5%, 79.5%, and 82.7% AS in trapezium, elliptical, and triangular model, respectively. In the range of 76.5%–79.5% the value of FFR for triangular and elliptical model was observed to be greater than 0.75, whereas the FFR value for trapezium model was lower than 0.75. At 82.7%, the triangular model shows FFR of 0.75 whereas trapezium and elliptical models show the FFR value of less than 0.75.

### 4.3. Pressure Drop Coefficient (CDP)

**Figure 6:**
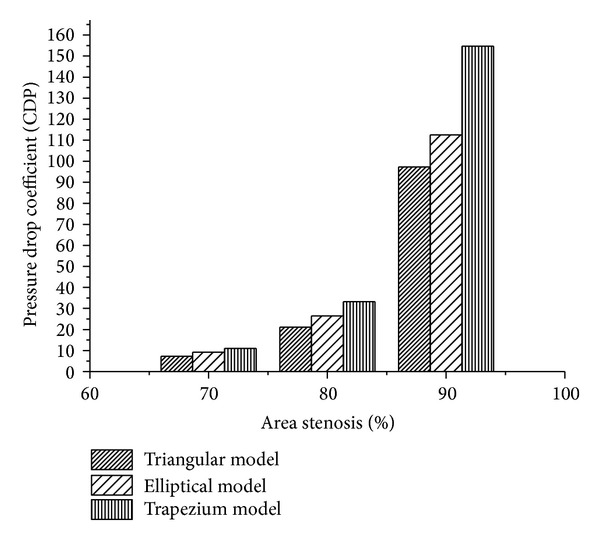
Variation of CDP with % AS in various shapes of models (triangular, elliptical, and trapezium).

The nonlinear increase in the value of CDP was observed for all the three different shapes of stenosis models as shown in Figure 6. For triangular model 3-fold increase in the value of CDP from 7.29 to 21.17 was observed in stenosis severity from 70% to 80% AS, whereas an increase in stenosis severity further to 90% AS elevated the CDP value by 4.6 times to 97.34. An elliptical model also shows a similar nonlinear trend such that 1.9-fold increase in CDP value from 9.19 to 26.45 was observed as the stenosis severity changed from 70% to 80 AS; this value further increased to 112.5 (4.3-fold) with an increase in the stenosis severity to 90% AS. For the case of trapezium model a 3-fold increase in the CDP value from 11.0 to 33.2 was observed in the stenosis severity from 70% to 80% AS, and further increase in stenosis severity to 90% AS elevated the CDP value by 154.7.

#### 4.3.1. Lesion Flow Coefficient (LFC)

**Figure 7:**
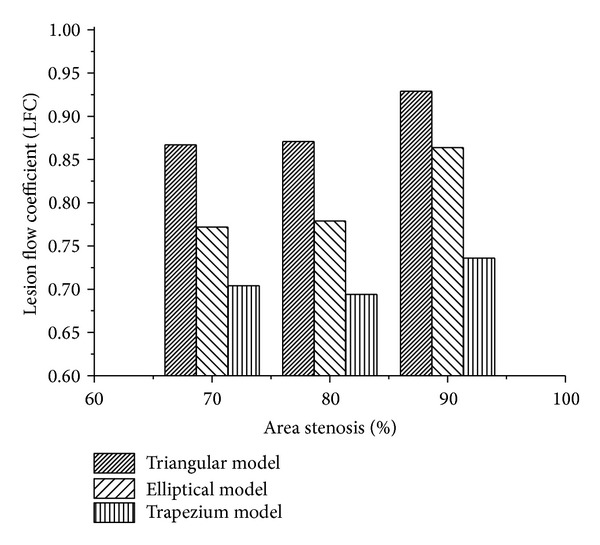
Variation of LFC with % AS in different shapes of models (triangular, elliptical, and trapezium).

Figure 7 depicts the variation of LFC for the different shapes of stenosis in different percentage AS. The value of LFC was found to be higher for the trapezium model than that of the other two models (triangular and elliptical). For the triangular shape stenosis, the increase in severity from 70% to 80% AS shows 1.1% increase in the LFC value. A further increase in 5.7% was observed for an increase in stenosis severity to 90% AS. However the elliptical model does not exhibit variation in the value of LFC (0.77) with corresponding increase in severity from 70% to 80% AS. For 90% AS the LFC increased to 11.6% (0.86). For the case of trapezium model the LFC was found to be the same (0.70) for both 70% and 80% and is increased to 4.2% (0.73) for 90% AS.

## 5. Discussion

The present study explains the effect of different shapes of stenosis models on the variation of the different diagnostic parameters, which otherwise have been reported by few other researchers [9, 11]. The primary objective of the study is to investigate the variation in the FFR, CDP, and LFC values for the given percentage area stenosis in the three different shapes of stenosis models.

To study the effect of different shapes of stenosis on the flow and pressure field, we have compared the pressure drop and hence the diagnostic parameters obtained for different shapes for the cases of 70%, 80%, and 90% percentage AS. With an increase in the stenosis increased pressure drop was observed in all the three models. The comparison of axial pressure drop $\Delta \tilde{p}$ in the models (triangular, elliptical, and trapezium) for a given percentage AS, the $\Delta \tilde{p}$ in the trapezium shape stenosis models was higher than in the other two models (triangular and elliptical) have been observed during a cardiac cycle. This is due to the effect of the stenotic shape (“shape-effect”), for triangular model after the convergent; stenosis starts diverging to maximum so that the flow would be maximum which results in less drop in pressure. In case of elliptical model a rounded surface affects the flow less in terms of localised losses of pressure and recirculation. Consequently, both triangular and semiellipse models represent a less-severe pathology than a trapezium, reducing the risks of deposit, setting, and enhancement of a stenosis. The trapezium shape consists of throat section after the convergent which significantly affects the pressure drop as compared to the other two models. This shows that the shape of the stenosis plays a very important role.

Figure 5 shows a significant variation in the FFR values in all the three different shapes of stenosis model. For AS < 76.5%, the FFR values for all the models (triangular, elliptical, trapezium) were well above the cut-off value of 0.75 and for AS < 79.5%, the FFR value for trapezium was observed below the cut-off value whereas the other two models (triangular and elliptical) show above the cut-off value of 0.75 which could lead to the misdiagnosis of stenosis severity. Similarly in the range of 79.5%–82.5% AS, the elliptical model and trapezium models were below the cut-off value of 0.75 whereas the triangular model shows FFR of 0.75, raising the potential of misdiagnosis.

For AS ≥ 82.7 the coronary interventional procedure could be carried out irrespective of the stenosis shape. Thus the variation in FFR in the region of 76.5–82.7% AS could lead to the misdiagnosis of intermediate stenosis to decide upon coronary intervention around the clinically used cut-off value of 0.75 if the decision is based only on angiography instead of the actual measurement of FFR. From the above discussion it is obvious that the shape of stenosis plays important role in evaluating functional significance of the stenosis severity.

We believe that apart from FFR, the diagnostic parameters CDP and LFC could provide significant knowledge in the estimation of functional severity of coronary stenosis.

The values of FFR, CDP, and LFC obtained from this simulation study for the trapezium model were compared with the previous work done by Konala et al. [14] in the rigid artery with rigid plaque model and are reported in Table 2.

From this study, it can be said that the different shapes of stenosis play a vital role in the FFR value in addition to the plaque size, position of the stenosis, curvature of artery, and its components. The variations in the diagnostic parameter due to the shape of stenosis might lead to misinterpretation in the evaluation of functional severity of intermediate stenosis.

The following are the limitations of study.

1. The selected CAD model has smooth surface. To know more accurate physiologic variation in pressure drop in the stenosed arteries, a realistic model should be considered.
2. The computational simulation does not exhibit the exact realistic physiologic situation due to the movement of the coronary wall during the cardiac cycle.
3. Our present model has straight rigid artery, but the real coronary geometry is curved and more complicated. However use of realistic coronary artery model and different enhanced models could be considered in the future study to overcome the shortcomings of the present model to analyse the cut-off value on FFR more accurately.

## 6. Conclusion

For a given percentage area stenosis, the different shapes of stenosis affect the intraluminal flow and hence the changes in diagnostic parameter FFR were observed in all the three models (triangular, elliptical, and trapezium). In clinical settings, due to the effect of shapes of stenosis, there is a possibility of misinterpretation of diagnosis on stenosis severity in the intermediate stenosis case. From the well-established cut-off value of FFR = 0.75 [14], we found a region of uncertainty of stenosis severity between 76.5% and 82.7% AS in a single vessel CAD by plotting a linear approximate correlation between FFR and % AS. In addition to the plaque size and its components, irregular shape of an artery and insertion of guide wire affect the FFR. We conclude that the shapes of stenosis are also one of the deciding factors that influence the value of FFR.

## Figures and Tables

**Table 1:** Dimensions of geometric shapes of stenosis. All the dimensions are in mm.

Area stenosis AS (%)	*r*_*d*_ (mm)	*r*_*m*_ (mm)	Triangular			Elliptical			Trapezium		
			*l*_*c*_ (mm)	*l*_*m*_ (mm)	*l*_*r*_ (mm)	*l*_*c*_ (mm)	*l*_*m*_ (mm)	*l*_*r*_ (mm)	*l*_*c*_ (mm)	*l*_*m*_ (mm)	*l*_*r*_ (mm)
70	1.5	0.82	5	—	5	—	—	—	3.5	3	3.5
80	1.5	0.67	5	—	5	—	—	—	3.5	3	3.5
90	1.5	0.47	5	—	5	—	—	—	3.5	3	3.5

**Table 2:** Comparison of diagnostic parameters for trapezium model with rigid artery with rigid plaque wall model reported by Konala et al. [14].

Konala et al. [14]				Trapezium model		
% Area stenosis	FFR	CDP	LFC	FFR	CDP	LFC
70	0.88	16.5	0.57	0.89	11.06	0.70
80	0.78	33.5	0.68	0.72	33.27	0.69
90	0.54	142.6	0.74	0.36	154.72	0.73
